# Physico-Chemical Responses of *Alstroemeria* spp. cv. Rebecca to the presence of Salicylic Acid and Sucrose in vase solution during postharvest life

**DOI:** 10.1186/s12870-024-04814-1

**Published:** 2024-02-19

**Authors:** Hanifeh Seyed Hajizadeh, Somayeh Bayrami Aghdam, Hadi Fakhrghazi, Sinem Karakus, Ozkan Kaya

**Affiliations:** 1 Department of Horticulture, Faculty of Agriculture, University of Maragheh, Maragheh, 55136 553 Iran; 2 Department of Horticultural Science and Agronomy, Science and Research Branch, Islamic Azad University, Tehran, Iran; 3 Çölemerik Vocational School, Hakkari University, Hakkari, 30000 Turkey; 4 Department of Biology, Faculty of Science and Art, Erzincan Binali Yıldırım University, Erzincan, 24002 Turkey; 5 Republic of Turkey Ministry of Agriculture and Forestry, Erzincan Horticultural Research Institute, 24060 Erzincan, Turkey; 6 Department of Plant Sciences, North Dakota State University, Fargo, ND 58102 USA

**Keywords:** Microbial contamination, Leaf and Petal senescence, Peroxidase, Vase life

## Abstract

**Background:**

The primary challenge in the cut flower industry, specifically in the postharvest phase, is the short vase life of flowers. This issue, along with early leaf yellowing and perianth abscission, significantly diminishes the economic value of flowers due to their accelerated senescence. To tackle this, we conducted a factorial experiment on *Alstroemeria* cv. Rebecca, utilizing a completely randomized design with three replications. In this experiment the effects of varying concentrations of Salicylic acid (SA) (0, 1.5, and 3 mM) and sucrose (SU) (0% and 3%) were investigated on the postharvest quality of leaves and florets, with systematic evaluations every three days throughout their vase life.

**Results:**

This experiment revealed that the specific treatment combination of 1.5 mM SA + 3% SU (T5) markedly improved various parameters, such as vase life, total chlorophyll content, membrane stability index, relative fresh weight, and water uptake of cut flowers. In our analysis, we observed that this preservative solution not only extended the vase life and enhanced water uptake but also effectively preserved total chlorophyll, mitigated the loss of fresh weight, and reduced membrane deterioration in petals. Additionally, our results showed an increase in the activities of catalase (CAT) and peroxidase (POD) enzymes, as well as total protein content, alongside a decrease in malondialdehyde (MDA) and hydrogen peroxide (H_2_O_2_) levels. Moreover, this study noted a decrease in microbial populations in solutions containing different concentrations of salicylic acid.

**Conclusions:**

Our research demonstrated that alstroemeria flowers maintained in a solution with 1.5 mM SA + 3% SU exhibited a significantly prolonged vase life of up to 21 days, in contrast to the 15 days observed in control flowers kept in water. These results are highly beneficial for manufacturers in the cut flower industry, as they provide a viable method to substantially extend the vase life of cut flowers. Such an enhancement in flower longevity can lead to increased market value and customer satisfaction. Furthermore, the reduction in flower senescence and decay rates can contribute to decreased waste and greater efficiency in cut flower distribution and sales, offering a substantial advantage to manufacturers in this competitive market. The extended vase life and reduced senescence observed in alstroemeria flowers treated with 1.5 mM SA and 3% SU are attributed to SA's role in enhancing endogenous defense responses and sucrose's function as an energy source, collectively improving water uptake, and delaying the natural decay process.

## Background

*Alstroemeria*, with its striking beauty and diverse colors, has firmly established itself as a cornerstone in the commercial ornamental cut flower industry. Its prominence is particularly noted in key markets such as the United States, the United Kingdom, and Japan, and its cultivation is now spreading across the globe. This expansion underscores the growing consumer demand and appreciation for Alstroemeria's unique aesthetic appeal [1]. A critical aspect in the post-harvest management of cut flowers, especially for species like Alstroemeria with their significant transpiration surfaces, is the effective management of water absorption and bacterial control in preservative solutions. This dual approach is not only crucial for maintaining the flowers' aesthetic quality but also plays a significant role in extending their vase life, a factor that directly impacts their economic value in the floriculture market. It's noteworthy that a substantial proportion, around 20–25%, of freshly harvested flowers lose their commercial viability during the processes of harvesting, packaging, transportation, and marketing, highlighting the challenges and economic implications in the cut flower supply chain [2].

Understanding the intricate biological and physiological responses of these cut flowers during the storage phase is pivotal for developing refined and effective post-harvest treatments. The senescence of cut flowers, a complex process, is influenced by three primary factors: the plant’s water relations and carbohydrate reserves, microbial activity in the storage environment, and the impact of ethylene, a known senescence-accelerating hormone [3, 4]. Ethylene's role, for instance, extends beyond merely triggering senescence; it's involved in the biochemical pathways that lead to cellular structure breakdown, such as lipid peroxidation, which ultimately results in petal loss—a critical concern in maintaining the commercial appeal of cut flowers [5]. Furthermore, the concentration of soluble carbohydrates in the petals is a critical determinant of the flower's lifespan. These carbohydrates are not just energy sources; they are integral in maintaining cellular structures, thereby influencing the rate and nature of senescence. This interrelation between soluble carbohydrate levels and senescence are particularly intriguing, as it varies significantly among different cultivars. For instance, while some cultivars may exhibit petal wilting that is relatively insensitive to ethylene, it is closely associated with their internal carbohydrate levels. This distinction is essential in devising targeted post-harvest treatments [6].

The process of petal senescence, which essentially marks the end of the flower's commercial life, is characterized by a series of physiological changes. These include decreases in fresh weight, protein content, and pigment vibrancy, ultimately leading to cell death and petal abscission [7]. Leaf senescence, on the other hand, presents a different set of challenges. It is often a consequence of reduced photosynthetic activity and chlorophyll depletion, triggered by factors such as light exposure and gibberellic acid levels. The metabolic shifts during this phase, particularly the heightened activity of proteases and increased chlorophyll degradation, are critical in understanding the overall senescence process in cut flowers [8]. Additionally, the complex interplay between various stress factors and the onset of leaf senescence has been a subject of extensive research. This stress accelerate physiological changes typically associated with senescence, such as chlorophyll depletion and nutrient concentration reduction, thus hastening the decline in visual and structural integrity of the leaves [9]. In this regard, Alstroemeria serves as an excellent model for exploring the molecular basis of leaf senescence, offering insights that can be applied to a broader range of floricultural species [10]. In specific varieties like *Alstroemeria* cv. Rebecca, the sensitivity to ethylene is particularly noteworthy. Studies have shown that treatments with ACC significantly increase petal abscission, suggesting a heightened responsiveness to ethylene and highlighting the need for careful management of ethylene levels during storage and transport [11].

The exploration of salicylic acid and sucrose in vase solutions represents a critical area of research within the floriculture industry, offering profound implications for extending the post-harvest longevity and aesthetic appeal of *Alstroemeria* flowers. Premature leaf yellowing is a significant concern that diminishes the ornamental value and marketability of cut flowers; hence, identifying effective strategies to counteract this process is of paramount importance. The potential of salicylic acid and sucrose to modulate plant physiological responses and delay senescence addresses a vital need for sustainable and cost-effective post-harvest treatments. This topic not only enhances our scientific understanding of plant senescence mechanisms but also promises substantial advancements in commercial floriculture, ensuring that Alstroemeria and similar species retain their beauty and vitality longer for consumers. Given these insights, the current study embarks on a comparative analysis of the effectiveness of salicylic acid and sucrose in altering physiological and biochemical parameters in alstroemeria. This research is particularly important as it explores the mechanisms through which these chemicals influence senescence in both leaves and petals. The findings from this study are anticipated to contribute significantly to the development of more effective post-harvest treatments. By understanding and manipulating the actions of SA and sucrose in vase solutions, it may be possible to mitigate premature leaf yellowing, thereby enhancing the ornamental value and marketability of *Alstroemeria* flowers. This research, therefore, holds substantial promise for both the scientific understanding of plant senescence and practical applications in the floriculture industry.

## Results and discussion

### Physiological traits

#### Vase life

**Fig. 1:**
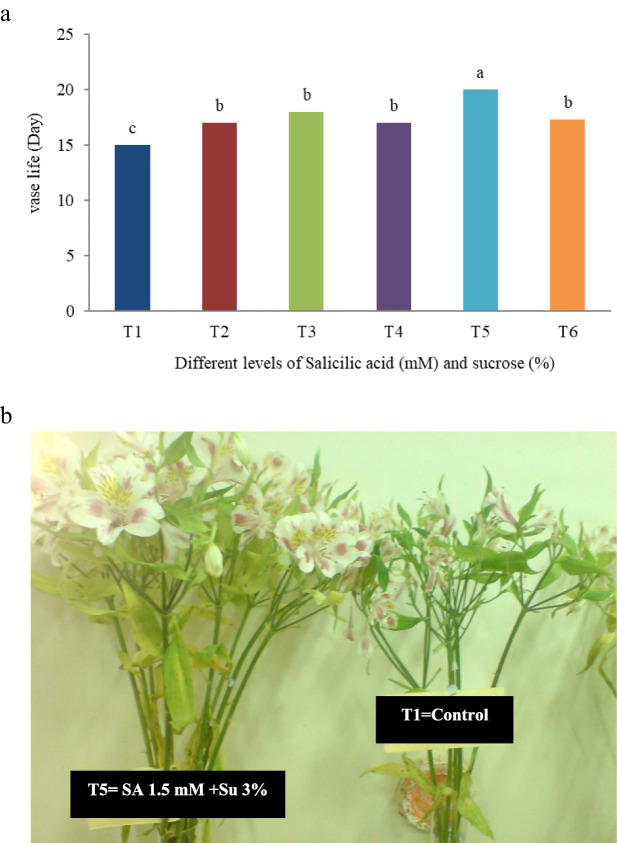
**a**) Effect of different preservative solutions on alstroemeria cv. Rebecca vase life; *T1*: Control, *T2*: SA 1.5 mM, *T3*: SA 3 mM, *T4*: SU 3%, *T5*: SA 1.5 mM + SU 3%, *T6*: SA 3 mM + SU 3%, and **b**) The comparison of vase life of T1 and T5 at 15^th^ day of *Alstroemeria* cv. Rebecca longevity

This research into the vase life of *Alstroemeria* cv. Rebecca, focusing on the impact of various vase solutions, has yielded significant insights into post-harvest cut flowers longevity. Central to our investigation was the hypothesis that the inclusion of specific chemical additives in vase solutions could substantially enhance the lifespan of cut flowers, compared to the conventional use of water. Our findings substantiated this hypothesis, demonstrating a considerable difference in lifespan between the control group, which was immersed in distilled water, and the groups exposed to alternative treatments. Specifically, our results highlighted that the treatment incorporating 1.5 mM Salicylic Acid (SA) and 3% sucrose significantly outperformed others, extending the cut flowers lifespan to 21 days. The mechanism of action underlying the efficacy of the salicylic acid and sucrose treatment is likely twofold: salicylic acid acts to delay senescence by modulating stress-related phytohormonal pathways and enhancing antioxidant defense systems, while sucrose provides an essential carbohydrate source that supports metabolic processes during the vase life. Together, these additives synergistically improve water retention, reduce ethylene production, and maintain cellular integrity, thereby extending the longevity of *Alstroemeria* cut flowers. This duration was notably longer than that observed in both the control group and other treatment groups, as clearly depicted in Fig. 1a. Moreover, our study provided a detailed comparative analysis of the vase life of Alstroemeria flowers in the control solution (T1) versus the SA and sucrose solution (T5) at the 15th day of vase life, effectively illustrated in Fig. 1b. This aspect of our research offered a visual affirmation of the quantitative data, bolstering our argument. Our research findings are in line with the studies conducted by Arrom and Munné-Bosch [12] on lilies, and Mei-Hua et al. [13] on gerberas, further validating our results. The consistency of these outcomes across various floral species underscores a pivotal botanical concept: the strategic application of certain chemical treatments in vase solutions can markedly prolong the vase life of cut flowers. The pronounced efficacy of the SA and sucrose combination in our study not only corroborates our initial hypothesis but also suggests a potential shift in the standard practices for post-harvest treatment of cut flowers, particularly for commercially valuable varieties like Alstroemeria. This insight holds considerable implications for the floriculture industry, pointing towards more effective and sustainable practices in flower preservation.

#### Flower relative fresh weight (RFW)

**Fig. 2:**
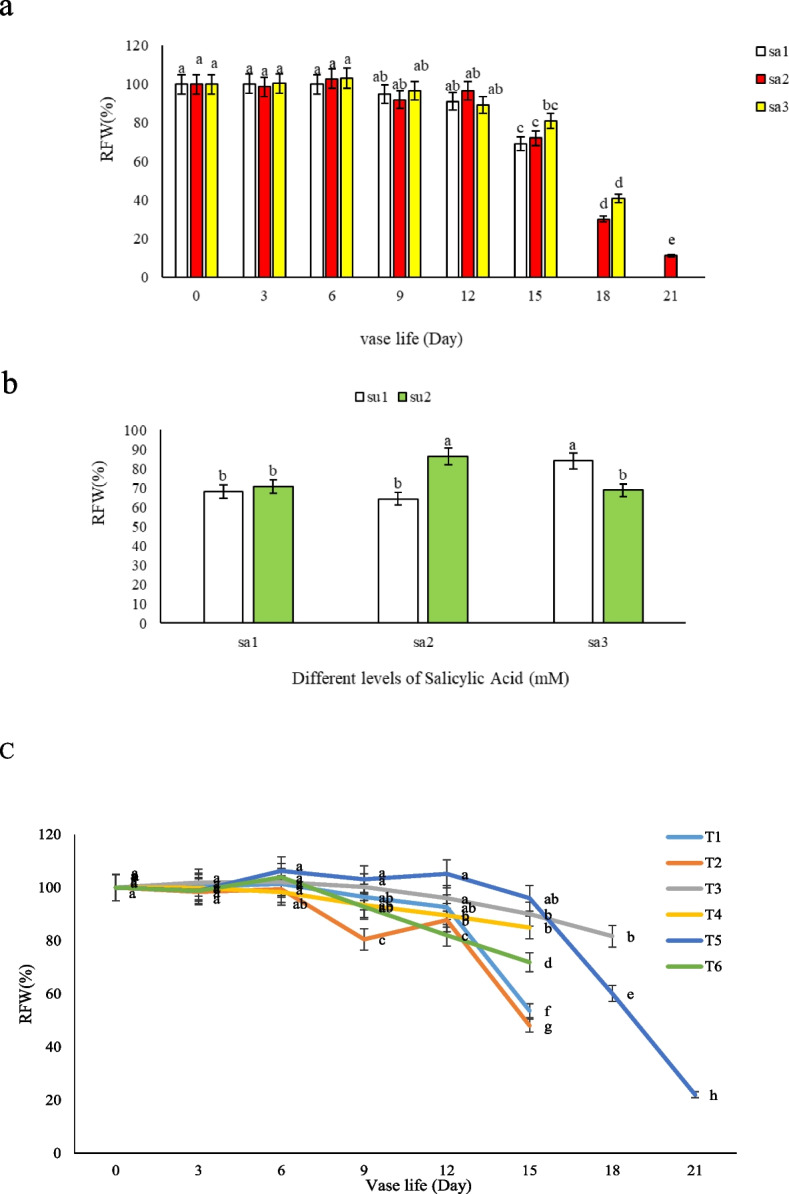
Effect of **a**) different salicylic acid concentrations (SA1 = 0, SA2 = 1.5, and SA3 = 3 mM), **b**) sucrose (SU1 = 0, and SU 2 = 3%), and **c**) different vase solutions on cut flower fresh weight during vase life of *Alstroemeria* cv. Rebecca; *T1*: Control, *T2*: SA 1.5 mM, *T3*: SA 3 mM, *T4*: SU 3%, *T5*: SA 1.5 mM + SU 3%, *T6*: SA 3 mM + SU 3%

**Fig. 3:**
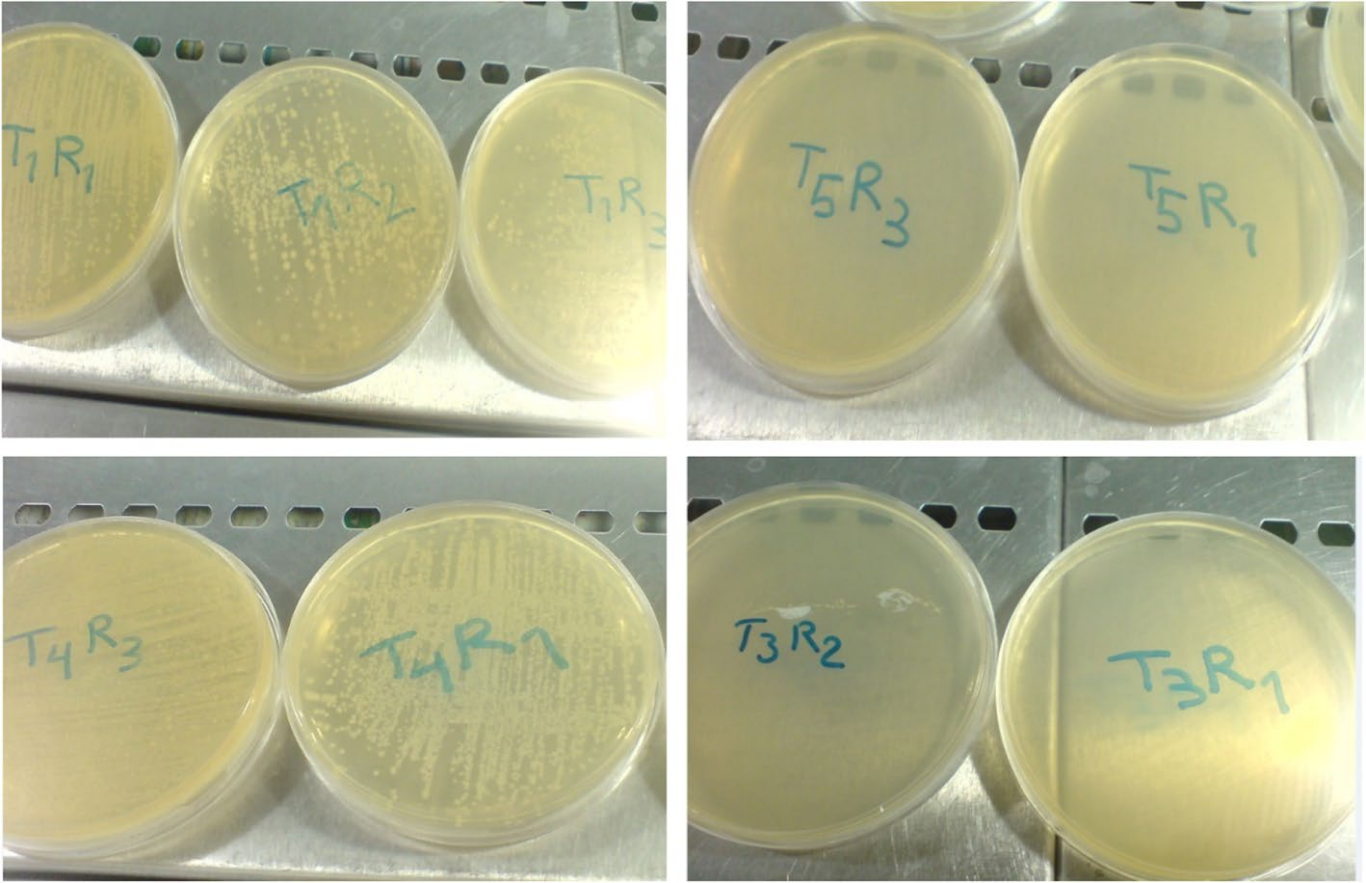
Cultivation of 50 µL of preservative solutions of *T1*, *T4* versus *T5*, and *T3* on the 15^th^ day of *Alstroemeria* cv. Rebecca vase life (*T1*: Control, *T3*: SA 3 mM, *T4*: SU 3%, *T5*: SA 1.5 mM + SU 3%)

This research on the vase life of Alstroemeria demonstrated a significant difference among treatments (*p* ≤ 1%), with the most notable findings observed in the treatment group T5, which maintained the highest relative fresh weight during vase life, particularly up to the 15^th^ day. According to Fig. 2c, it was evident that none of the treatments showed significant differences until the 3th day. However, a contrasting trend was observed in all treatments as depicted in Fig. 2a, where a temporary increase in relative fresh weight was noted on the 6^th^ day in cut flowers kept in T3 and T6 (Fig. 2c). Despite these fluctuations, the vase solution containing 3% sucrose + 1.5 mM salicylic acid consistently exhibited a higher relative fresh weight compared to other treatments and control. Furthermore, our analysis of the interaction between sucrose and salicylic acid (Fig. 2b) revealed that the combination of 1.5 mM SA with 3% sucrose resulted in the best performance in terms of flower fresh weight. Conversely, when the SA concentration was increased to 3 mM, sucrose did not exert a positive effect on flower fresh weight (Fig. 2b). These findings align with the research conducted by Hatamzadeh et al. [14], and Ezhilmati et al. [15] on gladiolus, underscoring the beneficial effects of certain chemical combinations on the preservation of cut flowers. Our study suggests that salicylic acid plays a crucial role in reducing evapotranspiration from the leaves and flowers, thereby preventing weight loss in cut flowers. SA reduces evapotranspiration in cut flowers by inducing stomatal closure, a mechanism that conserves water, maintaining flower weight and freshness. This process is likely bolstered by the presence of sucrose, which serves as a vital energy source for the flowers, sustaining metabolic activities that contribute to water retention and overall turgidity. Notably, the vase solution containing 3% sucrose + 1.5 mM salicylic acid maintained the maximum fresh weight (96%) compared to the control (53.64%) on the 15^th^ day. Overall, cut flowers kept in T5 and T3 vase solutions exhibited superior performance in maintaining relative fresh weight and extending vase life compared to other treatments and control. This enhancement can be attributed to the presence of salicylic acid, the acidic nature of the preservative solution, reduced microbial growth as had been seen in Fig. 3, and improved water absorption in the vessels leading to the aerial organs, leaves, and flowers. Our research contributes to the growing body of evidence supporting the use of specific chemical treatments in vase solutions to improve the longevity and quality of cut flowers, with particular emphasis on the synergistic effects of salicylic acid and sucrose.

#### Microbial contamination

Our research findings, as depicted in Fig. 3, revealed a significant reduction in the microbial population in vase solutions containing 1.5 mM salicylic acid + 3% sucrose (T5) and 3% salicylic acid alone (T3) by the 15^th^ day. In contrast, the control treatments employing tap water (T1) and 3% sucrose (T4) exhibited a substantially higher microbial population in the cultured media. This observation suggests that salicylic acid exerts a negative impact on microbial growth in the vase solutions of cut flowers. We hypothesize that salicylic acid may exert its antimicrobial effects by disrupting microbial cell membranes or interfering with their metabolic processes, thereby reducing the population in vase solutions. Additionally, salicylic acid could potentially enhance the plant's own defense mechanisms, making the environment less conducive to microbial growth. The implications of these results are particularly relevant in understanding the decay mechanisms in cut flowers. As identified by Gururani et al. [16], one of the primary causes of decay in cut flowers is the blockage of stem vessels due to microbial contamination or chemical residues, which impedes the stem's ability to absorb water. The presence of such microbial contamination in preservative solutions is a critical factor in the deterioration of cut flowers. Our study thus underscores the effectiveness of salicylic acid not only in enhancing the vase life of cut flowers by maintaining their fresh weight but also in mitigating microbial growth in vase solutions. These findings contribute to the broader understanding of post-harvest flower management and highlight the potential of using salicylic acid as a component in vase solutions to preserve cut flower quality by preventing microbial proliferation and ensuring efficient water uptake through the stem.

#### Leaf chlorophyll

**Fig. 4:**
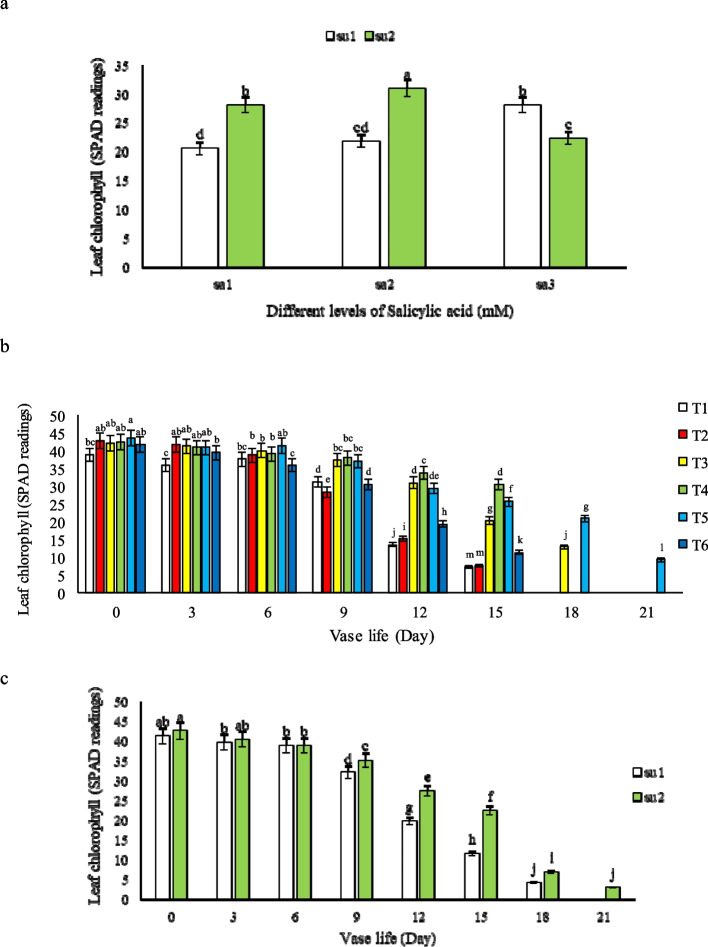
Effect of **a**) different salicylic acid (SA1 = 0, SA2 = 1.5, and SA3 = 3 mM) and sucrose concentrations (SU 1 = 0, and SU2 = 3%), **b**) different treatments (*T1*: Control, *T2*: SA 1.5 mM, *T3*: SA 3 mM, *T4*: SU 3%, *T5*: SA 1.5 mM + SU 3%, *T6*: SA3 mM + SU3%) and **c**) different sucrose concentrations (SU1 = 0, and SU2 = 3%) on *Alstroemeria* cv. Rebecca leaf chlorophyll indices during vase life

The results of salicylic acid and sucrose interaction on chlorophyll content of leaves had a significant difference (p ≤ 1%), as cut flowers kept in vase solutions T3 and T5 maintained the greenness of leaves better than the other treatments and control. In general, the effect of 1.5 mM SA was more than 3 mM salicylic acid specially in combination with 3% sucrose (Fig. 4a). Flowers kept in 1.5 mM SA + 3% SU had the maximum total leaf chlorophyll content (41.48) on the 6^th^ day (Fig. 4b). Although chlorophyll content in the leaves decreased during vase life in all treatments, in vase solution contains 3% sucrose, the mentioned reduction trend occurred at a slower rate so that the highest amount of chlorophyll reduction was related to flowers kept in 3% SU (T4) at 12 and 15 days after harvest. The preservation of leaf chlorophyll content may be hypothetically attributed to salicylic acid's role in upregulating genes associated with chlorophyll biosynthesis and its probable antioxidative action, which protects chlorophyll molecules from degradation. Furthermore, sucrose likely contributes to maintaining chlorophyll levels by serving as a carbon source, supporting the metabolic functions essential for maintaining chlorophyll synthesis and retention in the leaves.

However, they didn’t last long because of the vessels microbial blockage as illustrated in Fig. 3. It seems that the presence of 3% sucrose in T4 has a great role in preserving the chlorophyll of the leaves specially after 9^th^ day (Fig. 4c), and the effect of sucrose on leaf chlorophyll was much greater than the treatments that contain salicylic acid alone or in combination with sucrose. This can be clearly seen in treatments containing 3% sucrose + 1.5 mM salicylic acid (T5) and 3 mM salicylic acid (T3), which are long lasting treatments (Fig. 4b). Leaf yellowing started from 9^th^ days after harvest specially in control flowers and the one which kept in solutions having 1.5 mM SA and 3 mM SA + 3% sucrose.

#### MSI (%)

**Fig. 5:**
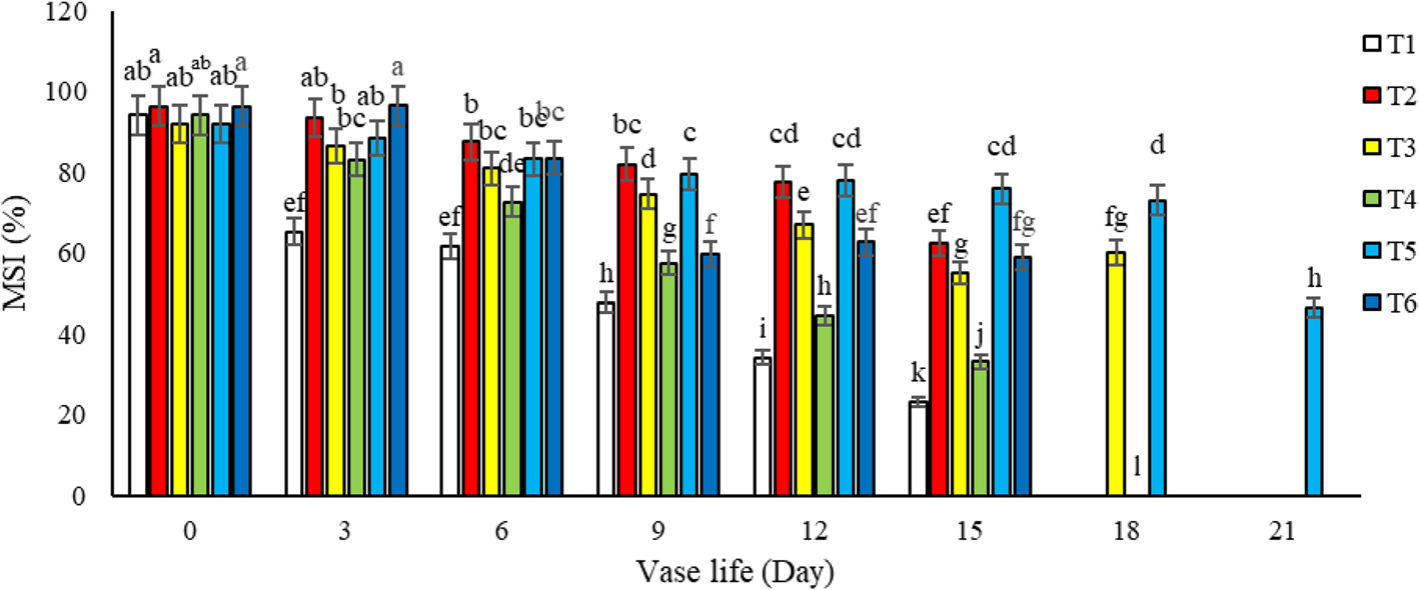
Effect of different treatments (*T1*: Control, *T2*: SA 1.5 mM, *T3*: SA 3 mM, *T4*: SU3%, *T5*: SA 1.5 mM + SU3%, *T6*: SA3 mM + SU3%) on *Alstroemeria* cv. Rebecca leaf MSI during vase life

Throughout the vase life of *Alstroemeria* cv. Rebecca, a decline in leaf membrane stability was observed across all treatments. However, treatments including T2, T3, T5, and T6, which featured salicylic acid either in isolation or in combination with sucrose, exhibited a notably higher membrane stability index in leaves by the 15^th^ day of longevity. This was in comparison to cut flowers maintained in water (control) and those in a vase solution containing only sucrose, as illustrated in Fig. 5. Particularly noteworthy was the performance of the vase solution containing 3% sucrose + 1.5 mM salicylic acid (T5), which surpassed other treatments in maintaining leaf membrane stability after day 15. Our research posits that treatments with varying concentrations of salicylic acid contribute to leaf membrane integrity by scavenging free radicals and inhibiting ethylene biosynthesis in cut alstroemeria flowers. The heightened MSI observed in leaves of cut Alstroemeria flowers treated with salicylic acid, particularly in the presence of sucrose, may be hypothetically linked to the upregulation of stress-responsive proteins and enzymes that bolster cell wall integrity and protect against lipid peroxidation. Additionally, the combined presence of sucrose could provide the necessary energy for the active repair of damaged membranes, further contributing to the maintenance of cellular stability and extending the decorative lifespan of the flowers. These actions are pivotal in delaying the senescence of the leaves. Our findings align with those of Hatemzadeh et al. [14], who observed similar effects on *Alstroemeria* cut flowers in their respective studies. Further, our results indicated that the preservative solution comprising 1.5 mM salicylic acid and 3% sucrose was more effective than other treatments in preventing membrane disintegration and electrolyte leakage from the leaves. The observed reduction in membrane stability during the vase life, attributed to the peroxidation of membrane lipids, is intricately linked to the aging process in the petals of cut flowers. This connection has been substantiated by the work of Bartoli et al. [17], who investigated the senescence of carnation petals and reported findings that support our observations. In general, our study highlights the beneficial role of salicylic acid, particularly when used in conjunction with sucrose, in preserving the structural integrity of leaf membranes in cut flowers, thereby extending their aesthetic and commercial viability. This insight contributes significantly to the floriculture industry's understanding of post-harvest flower preservation techniques.

#### Water uptake

**Fig. 6:**
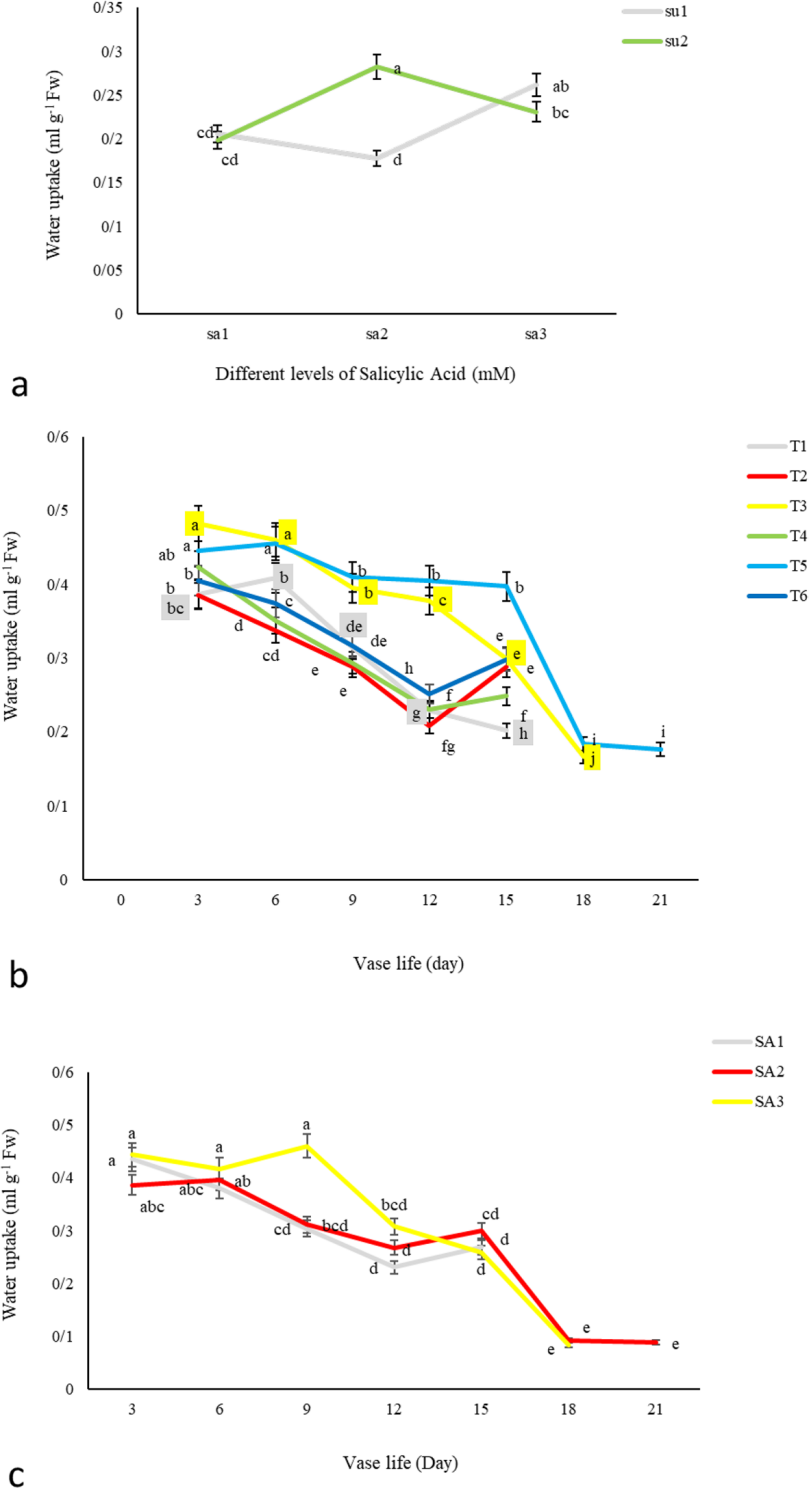
The effect of **a**) different SU (SU1 = 0, and SU2 = 3%) and SA (SA1 = 0, SA2 = 1.5, and SA3 = 3 mM) concentrations, **b**) different treatments (*T1*: Control, *T2*: SA 1.5 mM, *T3*: SA3 mM, *T4*: SU 3%, *T5*: SA1.5 mM + SU3%, *T6*: SA 3 mM + SU3%), and **c**) different SA concentrations (SA1 = 0, SA2 = 1.5, and SA3 = 3 mM) on *Alstroemeria* cv. Rebecca water uptake during vase life

Our study examined the interaction between sucrose and salicylic acid concentrations on water uptake in cut alstroemeria flowers, as depicted in Fig. 6a. The results indicated that a lower concentration of SA (1.5 mM) was more effective in facilitating water uptake compared to the higher concentration of SA (3 mM). Interestingly, at this lower concentration, the role of sucrose appeared to be negligible. Throughout the vase life, all treatments exhibited a decreasing trend in solution uptake from the first day until the end. However, this trend was significantly different in treatments 5 (3% sucrose + 1.5 mM SA) and 3 (3 mM SA), as shown in Fig. 6b. Treatment 5, in particular, demonstrated the highest solution absorption during longevity, outperforming both the other treatments and the control. Similarly, treatment 3 also showed better absorption rates than other treatments up to the end of the vase life of cut flowers. When comparing the effectiveness of SA concentrations, our findings revealed that 3 mM SA had a more pronounced effect than 1.5 mM SA up to 15 days after harvest, as illustrated in Fig. 6c. However, post-15 days, the efficacy of 1.5 mM SA, especially when combined with sucrose, was more pronounced (Fig. 6a). This observation suggests a synergistic effect between sucrose and SA at lower concentrations in enhancing water uptake. The enhanced water uptake observed with the combination of 1.5 mM salicylic acid and 3% sucrose may be attributed to the probable action of salicylic acid in improving xylem function and reducing vascular blockages, facilitating more efficient water transport. Additionally, the presence of sucrose could hypothetically improve the osmotic regulation within the flower stems, promoting better hydration and nutrient flow to the aerial parts. Our research also indicates that the reduced microbial accumulation in treatments T3 and T5, as shown in Fig. 3, corresponded with increased solution absorption in the xylem. Furthermore, there appears to be a direct correlation between vase life and water absorption in cut flowers. Cut flowers kept in vase solutions T3 and T5, which exhibited longer vase life, also demonstrated higher water uptake. This finding is in line with existing literature, which emphasizes the critical role of water uptake in extending the vase life of cut flowers [18]. It also reinforces the importance of selecting appropriate chemical treatments to enhance the longevity and quality of cut flowers, a key consideration in the floriculture industry.

#### Leaf H_2_O_2_

**Fig. 7:**
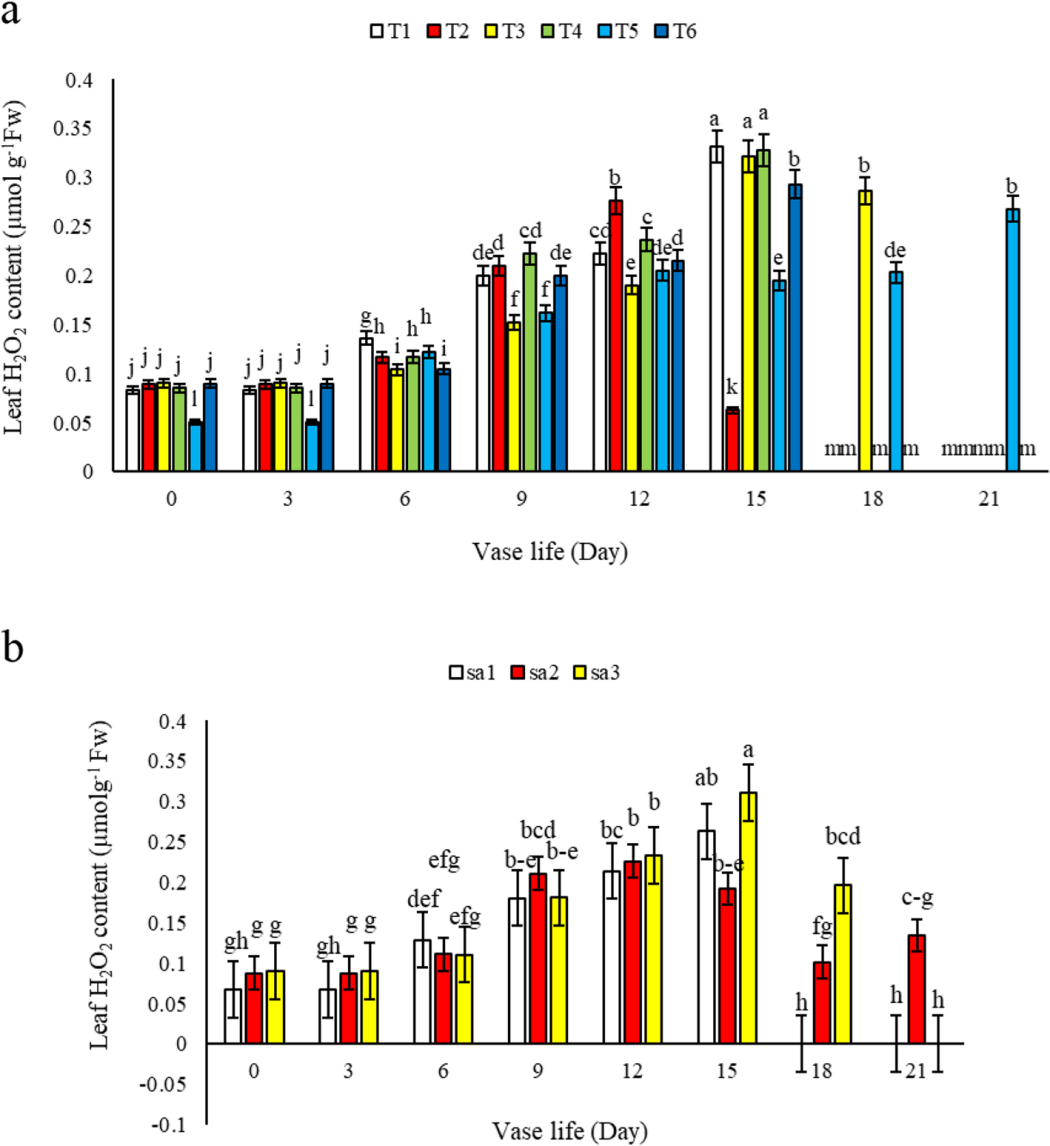
The effect of a) different treatments (*T1*: Control, *T2*: SA 1.5 mM, *T3*: SA 3 mM, *T4*: SU 3%, *T5*: SA 1.5 mM + SU3%, *T6*: SA 3 mM + SU3%) and b) different Sa concentrations (SA1 = 0, SA2 = 1.5, and SA3 = 3 mM) on leaf H_2_O_2_ content of *Alstroemeria* cv. Rebecca during vase life

In our analysis of leaf hydrogen peroxide (H_2_O_2_) content in alstroemeria, as shown in Fig. 7a, we observed an increasing trend from the 9th to the 15th day, followed by a subsequent decrease. This rise in leaf H_2_O_2_ content is indicative of escalated oxidative stress during this phase of the flower's vase life. Such stress contributes to increased lipid peroxidation and malondialdehyde levels, ultimately resulting in a decline in the membrane stability index and the senescence of the petals. Our results align with the findings of Chakrabarty et al. [5], who also reported similar trends in oxidative stress markers in cut flowers. Throughout the vase life, an upward trend in hydrogen peroxide content was noted across all treatments. However, vase solution T5 (1.5 mM SA + 3% sucrose), T3 (3 mM SA), T6 (3 mM SA + 3% sucrose), and T2 demonstrated higher leaf H_2_O_2_ content than the control, especially on the 15th day. This pattern underscores the impact of salicylic acid in the vase solution on oxidative stress markers (Fig. 7a). Notably, flowers in vase solutions T5 and T6, containing 1.5 and 3 mM SA along with 3% sucrose, exhibited the lowest H_2_O_2_ content compared to the control and other treatments. The minimal H_2_O_2_ levels were observed in flowers kept in 1.5 mM SA + 3% sucrose (Fig. 7b), suggesting an efficient regulation of antioxidant enzyme activities such as catalase in these treatments. The reduction in leaf H_2_O_2_ content observed in treatments containing 1.5 mM SA and 3% sucrose may be attributed to the enhanced activation of antioxidant defense mechanisms, including the upregulation of catalase and other antioxidative enzymes, which effectively scavenge reactive oxygen species. Moreover, the presence of sucrose in the vase solution might serve as an additional energy source, supporting the flower's metabolic processes to counteract oxidative stress and delay senescence. Our findings are consistent with the research of Hatemzadeh et al. [14], and Saeed et al. [19]. These studies collectively emphasize the role of salicylic acid and sucrose in modulating oxidative stress in cut flowers, thereby contributing to the mitigation of senescence processes. The results of our study highlight the importance of optimizing vase solution compositions to enhance the longevity and quality of cut flowers by regulating oxidative stress and associated biochemical parameters.

#### Leaf MDA

**Fig. 8:**
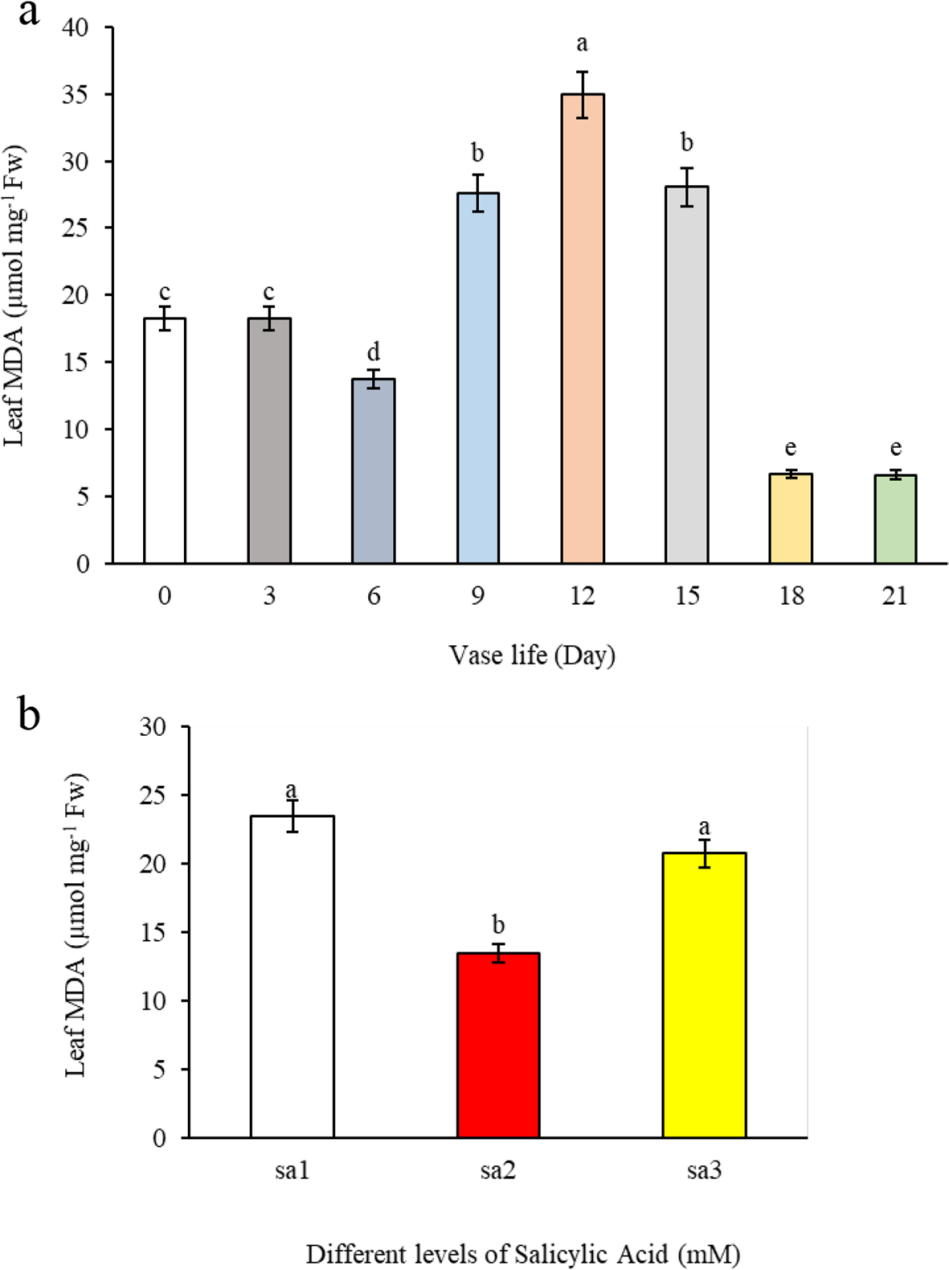
The effect of **a**) vase life, and **b**) different SA concentrations (SA1 = 0, SA2 = 1.5, and SA3 = 3 mM) on leaf MDA content of *Alstroemeria* cv. Rebecca

During the vase life of alstroemeria leaves, our study observed an increase in MDA content, indicative of lipid peroxidation, particularly peaking on the 12^th^ day. This trend suggests that the aging process of the leaf membranes commences around the 9^th^ day, reaching its zenith on the 12th day, as evidenced by the corresponding increase in hydrogen peroxide content (Fig. 8a) and the decline in chlorophyll content (Fig. 4a). Consequently, on the 15^th^ day, the leaf MDA content was observed to be higher than that at-harvest. Our findings corroborate with those of Nisar et al. [20], who reported similar patterns in lipid peroxidation during plant senescence. The observed decrease in lipid peroxidation past the 15th day could be attributed to the diminished quantity of lipids present in the leaf tissues. Figure 8b reveals that salicylic acid at a concentration of 1.5 mM was more effective in preventing malondialdehyde production compared to both the higher concentration of 3 mM salicylic acid and the control. This lower concentration of salicylic acid resulted in reduced MDA content, signifying its role in mitigating lipid peroxidation.

The lower concentration of salicylic acid (1.5 mM) may exert its protective effect against lipid peroxidation by enhancing the expression of antioxidative enzymes, thereby reducing the accumulation of malondialdehyde (MDA) and other oxidative stress markers. Additionally, salicylic acid could modulate ethylene perception and response pathways in cut flowers, minimizing ethylene's senescence-accelerating effects and thus contributing to the maintenance of cell membrane integrity over an extended vase life. The efficacy of salicylic acid in delaying senescence, as suggested by our results, aligns with the findings of Hassan and Ali [21]. It appears that the application of salicylic acid, potentially through reducing sensitivity to ethylene or diminishing its production, is particularly beneficial for Alstroemeria cut flowers, which are known to be ethylene-sensitive. This intervention thus offers a promising strategy for prolonging the freshness and aesthetic appeal of these flowers by mitigating the biochemical processes associated with senescence.

#### Petal MDA

**Fig. 9:**
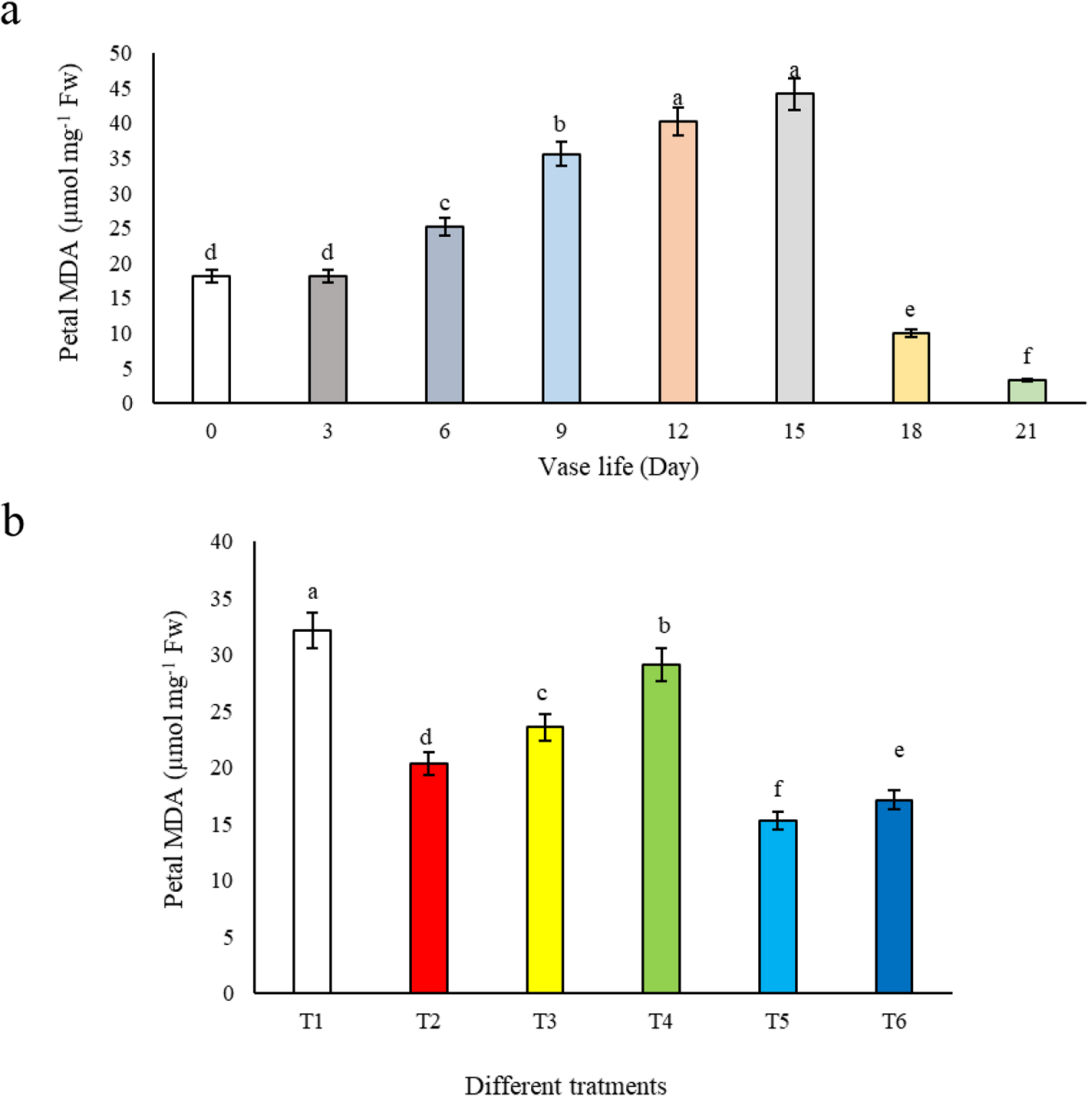
The effect of **a**) vase life, and **b**) different treatments (*T1*: Control, *T2*: SA 1.5 mM, *T3*: SA 3 mM, *T4*: SU3%, *T5*: SA 1.5 mM + SU3%, *T6*: SA 3 mM + SU3%) on petal MDA content of *Alstroemeria* cv. Rebecca

**Fig. 10:**
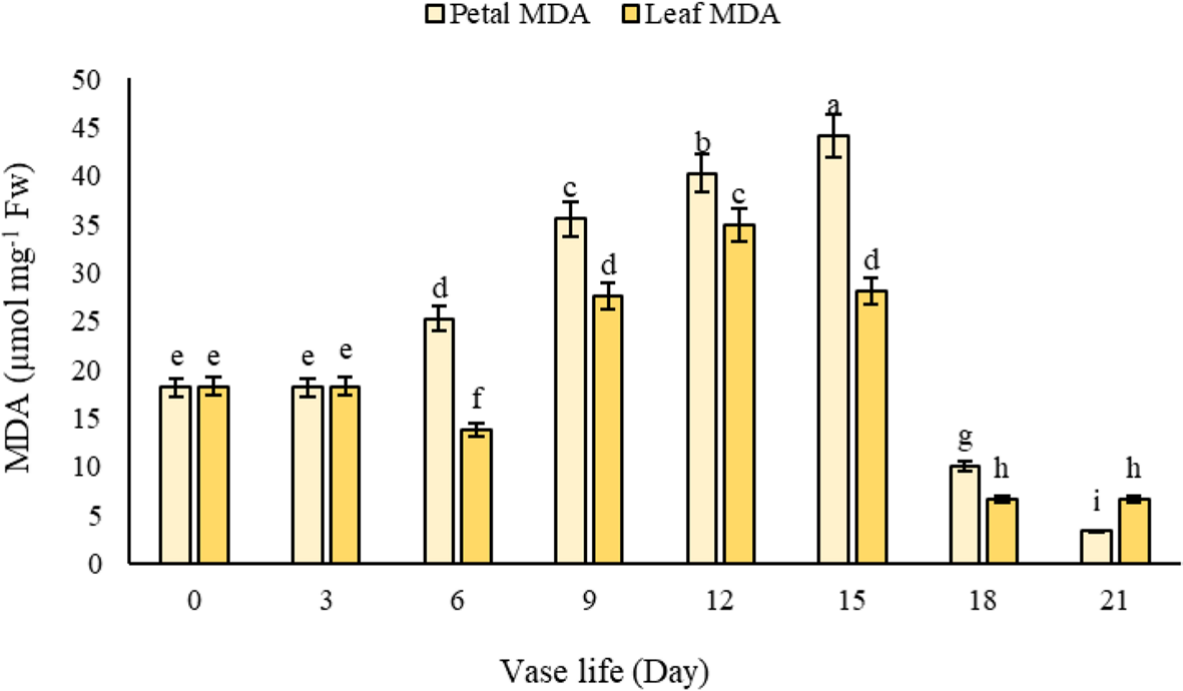
The effect of vase life on petal and leaf MDA content of *Alstroemeria* cv. Rebecca

Our investigation into the production of MDA in alstroemeria flowers revealed a progressive increase in MDA levels in petals throughout the vase life, with the highest production observed on the 15th day (Fig. 9a). Interestingly, flowers treated with T2, T3, T5, and T6 exhibited a significant difference compared to T4 and control treatments, displaying the lowest levels of lipid peroxidation due to reduced MDA production (Fig. 9b). In contrast, flowers maintained in tap water (T1) and those in 3% sucrose solution (T4) not only showed the highest MDA content but also the most pronounced petal deterioration (Fig. 9b). Our findings are in alignment with the research conducted by Hatamzadeh et al. [14], which also emphasized the beneficial role of salicylic acid in diminishing MDA content in both leaves and petals of cut flowers. This reduction in MDA levels is indicative of a lower rate of lipid peroxidation, a key factor in the senescence and deterioration of floral tissues. The significant decrease in MDA levels in petals treated with salicylic acid-based solutions (T2, T3, T5, T6) underscores salicylic acid's potential to mitigate oxidative stress by bolstering the antioxidative defense system, thereby reducing the rate of lipid peroxidation and consequent petal deterioration. This protective effect of salicylic acid might also involve the modulation of ethylene sensitivity or production in alstroemeria petals, further delaying the senescence process by maintaining cellular integrity and extending the visual appeal of the flowers. Furthermore, our comparative analysis of the impact of vase life on MDA content in both leaves and petals of alstroemeria flowers demonstrated that the peak of lipid peroxidation occurs at different times in these two organs. While the peak was observed on the 12^th^ day in leaves, it occurred on the 15^th^ day in petals (Fig. 10). This finding suggests that the onset of senescence occurs at varying times in different floral organs, starting approximately 3 days earlier in leaves. Our results corroborate those of Jędrzejuk et al. [22], who reported similar patterns of differential senescence timing in various floral organs. These insights highlight the complex nature of senescence in cut flowers and underscore the importance of considering the specific physiological responses of different floral parts when developing post-harvest treatments. Understanding these dynamics is crucial for the floriculture industry to optimize preservation methods and extend the aesthetic longevity of cut flowers like alstroemeria.

#### Leaf protein

**Fig. 11:**
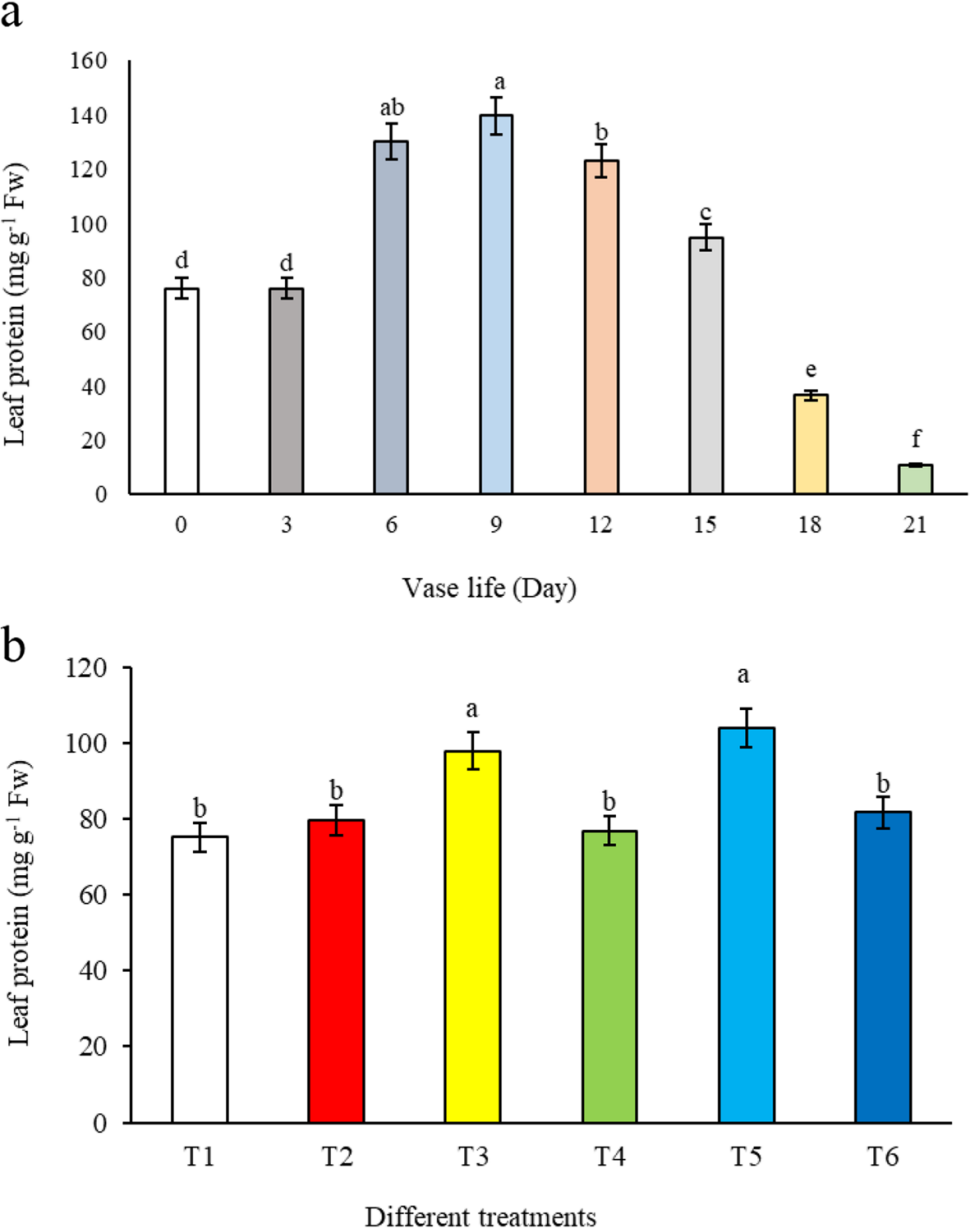
The effect of **a**) vase life, and **b**) different treatments (*T1*: Control, *T2*: SA 1.5 mM, *T3*: SA 3 mM, *T4*: SU3%, *T5*: SA 1.5 mM + SU3%, *T6*: SA 3 mM + SU3%) on leaf protein of *Alstroemeria* cv. Rebecca

In our study of alstroemeria cut flowers, we observed a consistent decrease in leaf protein content throughout the vase life, as depicted in Fig. 11a. This decline in protein levels is closely associated with the process of leaf senescence. One probable explanation for this phenomenon is that the deterioration of the cytoplasmic bilayer membrane, which occurs in tandem with aging, leads to a loss of membrane protein functionality. This correlation between senescence and protein degradation aligns with the findings of Gerailoo and Ghasemnezhad [23], who also reported similar trends in their respective studies. Remarkably, the protein content in *Alstroemeria* flowers treated with 1.5 mM salicylic acid combined with 3% sucrose was found to be higher than in the control group and other treatments (Fig. 11b). This suggests that the application of salicylic acid, particularly when used in conjunction with sucrose, plays a role in mitigating protein loss during the senescence process.

The preservation of higher protein content in *Alstroemeria* flowers treated with 1.5 mM salicylic acid and 3% sucrose may be attributed to salicylic acid's ability to modulate stress response pathways, reducing protein degradation associated with senescence. Additionally, sucrose could act as an energy source, supporting cellular activities that prevent the breakdown of proteins and maintain membrane integrity during the senescence process. Such an effect can be instrumental in preserving the structural and physiological integrity of the leaves, thereby potentially extending the aesthetic appeal and commercial value of cut alstroemeria flowers. Our results contribute to the broader understanding of post-harvest physiology in cut flowers, particularly highlighting the importance of optimizing vase solution compositions to counteract the natural senescence processes. The preservation of leaf protein content is a key factor in maintaining the overall health and appearance of cut flowers, making the insights from this study valuable for the floriculture industry's post-harvest management practices.

#### Petal protein

**Fig. 12:**
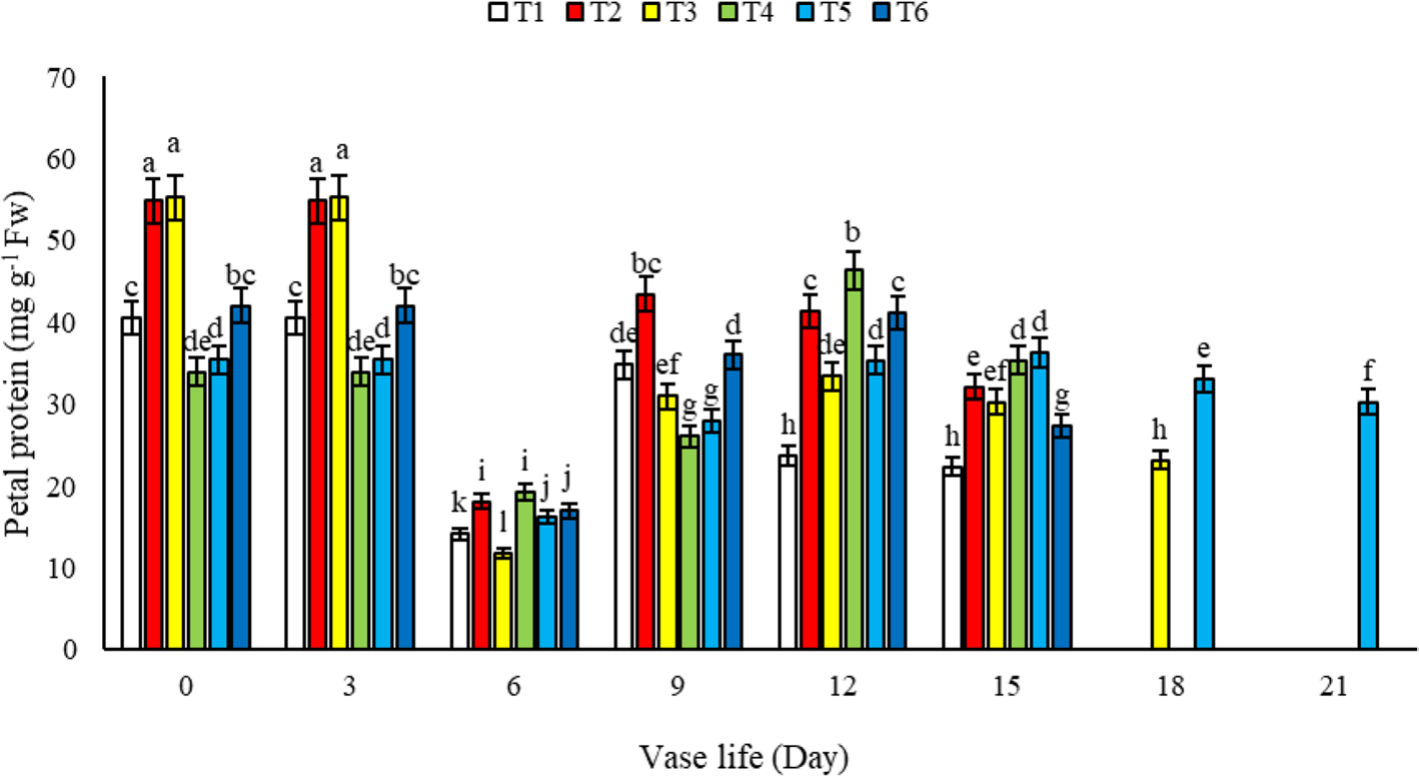
The effect of different treatments (*T1*: Control, *T2*: SA 1.5 mM, *T3*: SA 3 mM, *T4*: SU3%, *T5*: SA 1.5 mM + SU3%, *T6*: SA 3 mM + SU3%) on petal protein of *Alstroemeria* cv. Rebecca during vase life

During the vase life of alstroemeria cut flowers, our study observed a dynamic pattern in petal protein content. Specifically, in flowers treated with T2, T3, and T5 solutions, there was an initial increase in protein levels up to the 3^th^ day, followed by a decline starting on the 6^th^ day, and then a subsequent increase. Notably, the protein content in the petals of flowers maintained in a vase solution containing 1.5 mM salicylic acid (SA) + 3% sucrose (T5) was significantly higher than that in other treatments and control by the 15^th^ day (Fig. 12). This finding underscores the efficacy of this particular vase solution in preserving petal protein content over an extended period. The trend we observed in petal protein content aligns with the research conducted by Stead and Van Doorn [24], who also noted similar patterns in protein dynamics in cut flowers. Our results suggest that the incorporation of salicylic acid, especially when combined with sucrose, plays a crucial role in maintaining protein integrity in the petals.

The observed preservation and subsequent increase in petal protein content in treatments with 1.5 mM salicylic acid and 3% sucrose may be attributed to the role of salicylic acid in activating protective mechanisms against proteolytic activity, while sucrose potentially provides the necessary energy for protein synthesis and repair processes in the petals. Furthermore, this combination may enhance the expression of genes involved in protein stabilization, contributing to the maintenance of cellular functions and structural integrity of the petals during senescence. This is particularly significant as proteins are vital for various physiological functions and structural integrity of the petals, which are key factors in the overall aesthetic quality of cut flowers. The preservation of petal protein content not only contributes to maintaining the structural integrity and visual appeal of the flowers but also potentially extends their marketable lifespan. Our study adds to the growing body of evidence supporting the use of specific chemical treatments in vase solutions to improve the longevity and quality of cut flowers, with a particular emphasis on the synergistic effects of salicylic acid and sucrose. These insights are invaluable for the floriculture industry, offering practical solutions for enhancing the post-harvest life of cut flowers like alstroemeria.

#### Peroxidase activity in leaf and petal

**Fig. 13:**
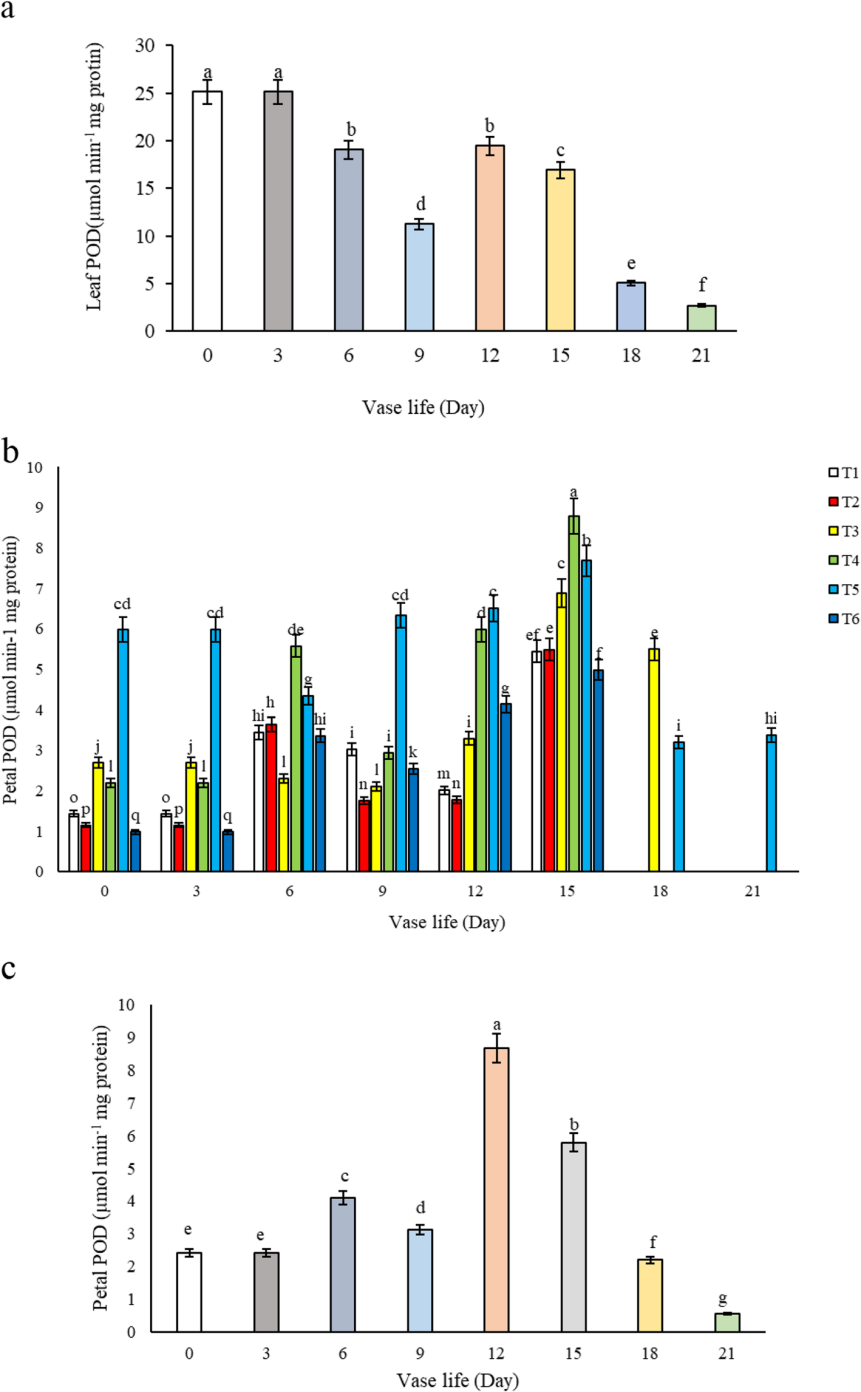
Effect of **a**) vase life on leaf POD activity, **b**) different treatments (*T1*: Control, *T2*: SA 1.5 mM, *T3*: SA 3 mM, *T4*: SU3%, *T5*: SA 1.5 mM + SU3%, *T6*: SA 3 mM + SU3%) during vase life, and **c**) vase life on petal POD activity of *Alstroemeria* cv. Rebecca

In our analysis of leaf peroxidase (POD) activity in alstroemeria flowers, as presented in Fig. 13a, we observed a general decreasing trend in enzyme activity over the vase life, with temporary increases noted on the 12^th^ and 15^th^ days in comparison to the 6^th^ and 9^th^ days. This pattern suggests that the peak of leaf senescence around the 12^th^ day is associated with a surge in POD activity, which coincides with the increased production of H_2_O_2_. POD, being one of the key antioxidant enzymes, plays a critical role in directly eliminating free radicals that contribute to oxidative stress. Our findings are consistent with those reported by Sharma et al. [25], who observed similar trends in POD activity in relation to senescence and oxidative stress. When examining the effect of different treatments on POD activity in alstroemeria petals during vase life, it was evident that flowers treated with T5 (1.5 mM salicylic acid + 3% sucrose) and T3 (3 mM salicylic acid) vase solutions exhibited the highest enzyme activity by the 15th day compared to the control and other treatments, as indicated in Fig. 13b. Also, the highest petal POD activity was observed on 12^th^ day after harvest as depicted in Fig. 13c. The elevated POD activity in vase solutions containing salicylic acid is indicative of enhanced scavenging of free radicals, which in turn contributes to the prolongation of the vase life of cut flowers.

The observed fluctuations in peroxidase (POD) activity, with peaks aligning with the senescence timeline, suggest that POD's role in mitigating oxidative stress becomes increasingly crucial as senescence progresses, particularly in response to the elevated levels of H_2_O_2_. The significant increase in POD activity in treatments containing salicylic acid, such as T5 and T3, may be due to salicylic acid's ability to enhance the plant's antioxidative response, promoting the activity of POD and other antioxidative enzymes to neutralize free radicals effectively. Our study underscores the importance of salicylic acid in modulating antioxidant enzyme activities, such as POD, in cut flowers. By enhancing these natural defense mechanisms, salicylic acid in vase solutions can effectively mitigate oxidative stress, thereby improving the overall health and longevity of cut alstroemeria flowers. These insights are valuable for floriculture practices, offering a potential strategy to extend the commercial viability and aesthetic appeal of cut flowers through targeted post-harvest treatments.

#### Catalase activity in leaf and petal

**Fig. 14:**
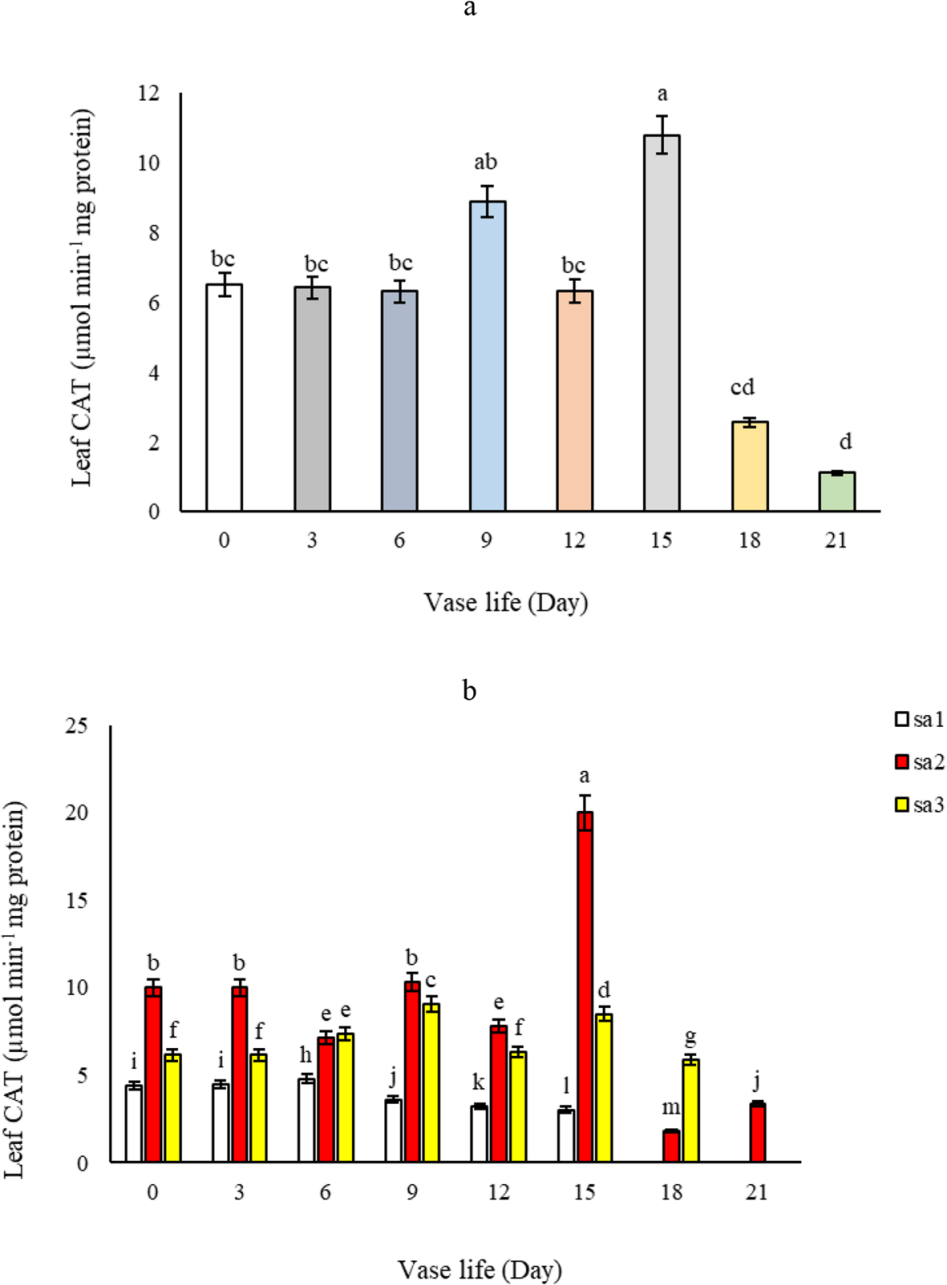
Effect of **a**) vase life, and **b**) different Sa concentrations (SA1 = 0, SA2 = 1.5, and SA3 = 3 mM) on leaf catalase activity of *Alstroemeria* cv. Rebecca during vase life

**Fig. 15:**
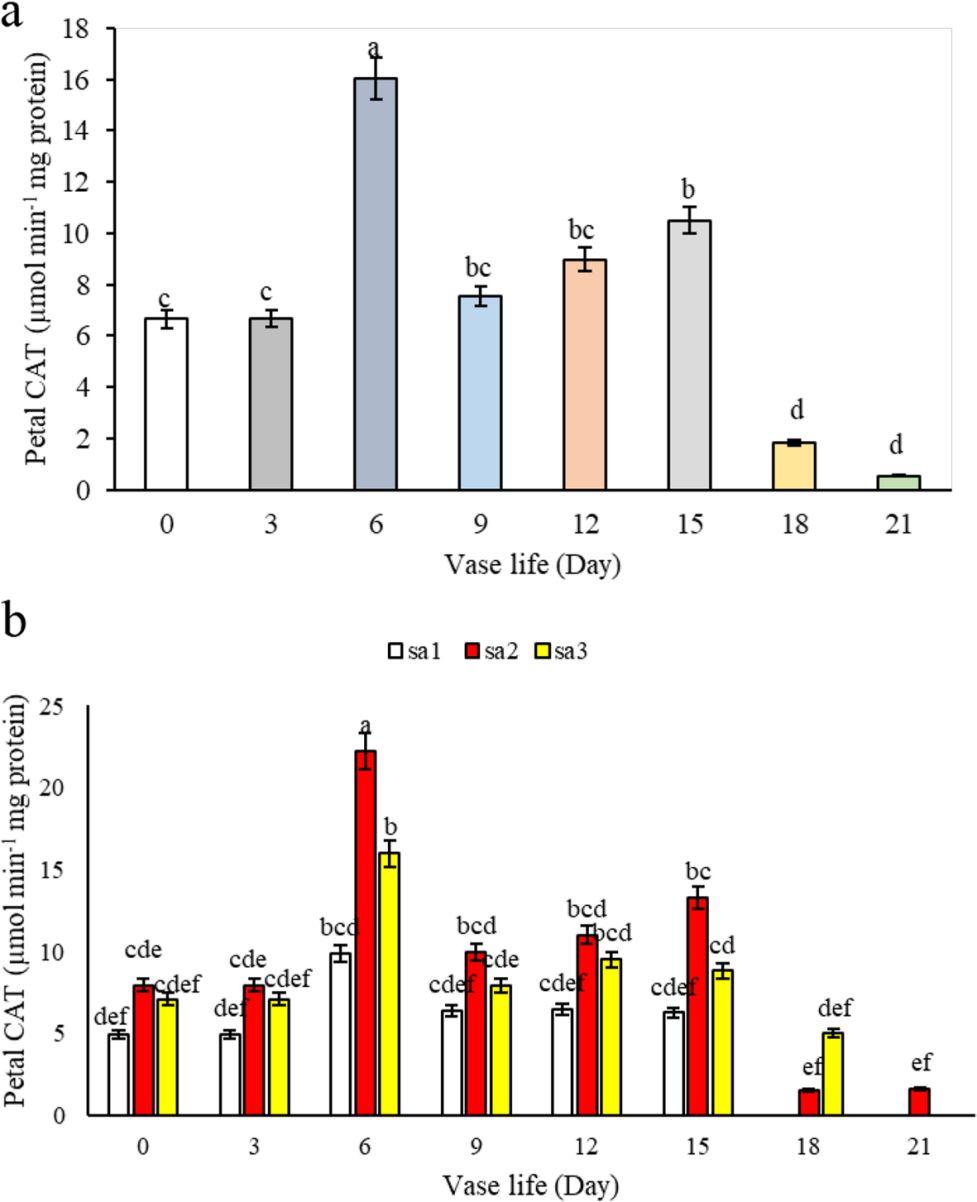
Effect of **a**) vase life, and **b**) different SA concentrations (SA1 = 0, SA2 = 1.5, and SA3 = 3 mM) on petal catalase activity of *Alstroemeria* cv. Rebecca during vase life

During the vase life of alstroemeria flowers, our study tracked the activity of catalase, an important antioxidant enzyme. As depicted in Fig. 14a, catalase activity generally exhibited a decreasing trend. However, a notable increase was observed from day 6 to day 15, followed by a decline to the lowest value on day 21. This transient increase is likely attributable to the natural activation of the plant’s antioxidant defense system, which functions to eliminate harmful free radicals and mitigate oxidative stress. These observations are in line with the findings of Chakrabarty et al. [5], who reported similar patterns in catalase activity. Regarding the impact of salicylic acid, different concentrations significantly influenced catalase activity. Flowers treated with 1.5 mM salicylic acid demonstrated the highest enzyme activity in leaves throughout the vase life (Fig. 14b), surpassing both the control and other concentrations of salicylic acid. The transient increase in catalase activity observed from day 6 to day 15 may reflect the plant's adaptive response to oxidative stress, where salicylic acid at a concentration of 1.5 mM enhances the efficiency of this antioxidant defense mechanism. This activation likely helps in delaying cellular damage and senescence by efficiently neutralizing harmful oxygen species, thereby contributing to the prolonged vase life and improved health of *Alstroemeria *flowers. This suggests that salicylic acid, particularly at this concentration, effectively scavenges free radicals, thereby slightly delaying the onset of wilting and maintaining membrane stability. This correlation supports the results on *Alstroemeria* presented by Hatamzadeh et al. [14]. In petals, catalase activity also followed a decreasing trend during vase life, with the exception of a temporary increase on day 6 across all treatments, as shown in Fig. 15a. The application of different levels of salicylic acid resulted in higher catalase activity in petals at the 1.5 mM concentration on the 6^th^ day of vase life compared to the control and other treatments. This pattern persisted until the end of the vase life, with the 1.5 mM concentration remaining more effective than the control and the 3 mM salicylic acid concentration, though both concentrations were more effective than the control (SA = 0) (Fig. 15b). The process of aging and degradation of petals appears to initiate slowly from day 6 post-harvest, coinciding with the observed increase in catalase activity for the removal of H_2_O_2_. In the context of wilting in cut flowers, where oxygen free radicals play a pivotal role, antioxidant enzymes like catalase are crucial in extending vase life by neutralizing these radicals. This is supported by earlier studies by Alaey et al. [26] who studied similar phenomena in rose cut flowers. The increased catalase activity may be attributed to the reduction of protein content in petals following flower opening and the onset of senescence.

## Conclusion

In our research, *Alstroemeria* cv. Rebecca cut flowers demonstrated the longest vase life, highest chlorophyll content, optimal relative fresh weight, enhanced water uptake, and increased membrane stability index when kept in a vase solution containing 1.5 mM salicylic acid (SA) and 3% sucrose. These conditions also resulted in the lowest microbial population compared to the control and other treatments. Our findings also revealed that flowers in 3 mM SA achieved a similar maximum vase life of about 21 days. A comparison of SA concentrations in our study showed that 1.5 mM SA led to the highest catalase activity and the least lipid peroxidation in leaves. Our observations indicated that leaf yellowing in *Alstroemeria* cv. Rebecca cut flowers started from the 9^th^ day after harvest, preceding flower senescence. However, our results highlighted that the combination of sucrose with 1.5 mM SA was more effective than 3 mM SA alone. Further analysis in our research showed that flowers in a vase solution with 1.5 mM SA and 3% sucrose had the highest protein content and POD activity in petals, and the lowest H_2_O_2_ content and lipid peroxidation. Generally, from our study's evaluation of leaf chlorophyll, MDA, and H_2_O_2_ content, it was concluded that petal senescence begins at least 6 days after the leaves. For producers, the implications of our findings are significant, offering extended shelf life and improved aesthetic quality, which can enhance marketability and consumer appeal. In future research, investigating the potential synergistic effects of different compounds with SA and sucrose, and fine-tuning their concentrations for various cut flower types, may yield further advances in prolonging cut flower longevity and quality.

## Materials and methods

### Plant material and treatments

*Alstroemeria* cv. Rebecca flowers were harvested at their commercial maturity stage, identified by the coloring of the buds and the imminent opening of the oldest buds [14], from a local commercial greenhouse (Pakdasht, Tehran, Iran). Following harvest, the cut flowers were transported to the laboratory. Here, the stems were trimmed to a length of 45 cm under water to prevent embolism. The cut stems were then placed into 1 L glass flasks, each containing 0.25 L of tap water. This setup served as the control treatment (T1). Additional treatments involved different preservatives containing different levels of Salicylic acid (0, 1.5, and 3 mM) and sucrose (0, and 3% w/v) including: T2 comprised 1.5 mM salicylic acid (SA), T3 contained 3 mM SA, T4 included 3% sucrose, T5 was a combination of 1.5 mM SA and 3% sucrose, and T6 consisted of 3 mM SA and 3% sucrose. All treated cut flowers were maintained under consistent conditions, at a temperature of 20 ± 2 °C and exposed to 12 h of fluorescent lighting, providing 850 lx illumination from cool-white, fluorescent lamps. The flowers' physiological and biochemical characteristics were assessed every three days until the end of their vase life.

### Measured characteristics

#### Vase life

The evaluation of vase life for the Alstroemeria cut flowers began from the day the spikes were transferred to their respective keeping solutions. Vase life was determined to end when the flowers had lost approximately 50% of their ornamental or display value. This loss of value was characterized by changes such as leaf color alteration, petal wilt, and loss of turgidity. Specifically, the end of an individual Alstroemeria cut flower stem's vase life was marked by visible signs of either 50% leaf yellowing or 50% petal abscission [14].

#### Relative fresh weight (RFW)

The relative fresh weight of the flowers was accurately measured using a digital scale with a precision of 0.01 g at two key points: initially, at the start of the experiment and before the stems were placed in their respective solutions, and subsequently at regular intervals throughout the duration of the experiment. To calculate the relative fresh weight of each flowering branch, the measurements were converted into a percentage value. This calculation was performed using Eq. (1) [14].

1 $${\text{RFW}}=({\text{Wt}}/{\text{Wt}}=0)\,\times\,100$$

#### Microbial contamination

To assess microbial contamination in the vase solutions, 1 ml of each solution was taken and diluted to a 10^^4^ concentration. These samples were then cultured on nutrient agar media. This process was conducted on the 15th day of the vase life and was replicated three times. After culturing, the samples were incubated at 37ºC for 48 h. The number of microorganisms present in the preservative solutions was determined by counting the former colony units post-incubation. These were recorded as colony-forming units per milliliter (CFU mL^−1^), as described by Seyed Hajizadeh et al. [27].

#### Leaf chlorophyll index

The assessment of leaf chlorophyll index in Alstroemeria flowers was performed using a portable chlorophyll meter, specifically the SPAD model from Konica Minolta. Measurements were taken every three days, starting from the first day of treatment application. For each measurement, two fully expanded leaves were selected, and the chlorophyll index was expressed as SPAD reading values, following the methodology outlined by Unal et al. [28].

Membrane Stability Index (MSI). This parameter was studied to determine the stability of the petal cell membrane. For this, 0.1 g of petals were weighed and cut into similar sizes. These petal samples were then placed in two sets of test tubes, each containing 10 mL of distilled water. The electrical conductivity of each set was measured after incubation at two different temperatures: first at 40 °C for 30 min (C1), and then at 100 °C for 15 min (C2). The electrical conductivity was measured using EC meters (Jenway model, UK), as per the methodology described by Almeselmani et al. [29]. The Membrane Stability Index was then calculated using formula (2);

2 $$\mathrm{MSI\,}(\mathrm{\%})=\,[1\,-\,({\text{C}}1\,/\mathrm{\,C}2)]\,\times\,100$$

#### Water uptake

The application of preservative solutions was diligently monitored and evaluated at three-day intervals after the flowers were transitioned from the storage buckets to the display vases. This meticulous assessment followed the protocol outlined by He et al. [30], involving the measurement of solution uptake, visual examination for signs of senescence or microbial growth in the solution, and recording any physiological changes in the flowers, such as petal wilting or leaf yellowing. The frequency of these evaluations ensured a comprehensive understanding of the preservative solutions' effectiveness over time, contributing to optimized post-harvest management practices. The results were expressed in ml g^−1^ FW.

#### Hydrogen peroxide (H_2_O_2_)

The H_2_O_2_ content in Alstroemeria petal was determined according to the protocol established by Liu et al. [31]. In this procedure, 0.5 g of petal samples were ground in liquid nitrogen with a potassium phosphate buffer (pH 6.8). The resulting extracts were centrifuged at 7000 rpm for 25 min at 4 °C. Then, 100 µL of the supernatant was mixed with 1 mL of xylenol solution. After a 30-min reaction time, the absorbance of the mixture was measured at 560 nm using a Shimadzu spectrophotometer. The results were expressed in μmol min^−1^ mg^−1^ protein FW.

Malondialdehyde (MDA). MDA levels were assessed as a 2-thiobarbituric acid (TBA) reactive metabolite, following the method described by Zhang et al. [32]. For this, 1.5 mL of extraction was mixed with 2.5 mL of 5% TBA in 5% trichloroacetic acid. The reaction mixture was heated at 95 °C for 15 min and then quickly cooled. Post centrifugation at 5000 rpm for 10 min, the absorbance of the supernatant was read at 532 nm. The results were expressed in μmol min^−1^ mg^−1^ protein FW.

Protein content. For determining the protein content in the petals, the Bradford [33] method was utilized. This process involved adding 100 µl of the extracted solution into a glass tube, followed by 5 ml of Bradford reagent. After a 20-min reaction period, the absorbance of the mixture was measured at 595 nm, using a blank sample for comparison. The blank was prepared by mixing 100 µl of Tris–HCl buffer with 5 mL of Bradford reagent. The concentration of the extracted protein was then calculated using a standard curve. The results were expressed in μmol min^−1^ mg^−1^ protein FW. Peroxidase activity (POD). POD was measured following the protocol by Abeles and Biles [34]. The assay involved adding 4 mL of 0.4 M sodium acetate buffer, 5 mL of 3% H_2_O_2_, and 0.2 mL of 50% benzidine 2-methanol to each glass tube. Then, 0.2 mL of the extract was added, and the absorbance was read immediately at 530 nm. For the blank sample, 0.2 mL of Tris–HCl buffer was used instead of the extract. The results were expressed in μmol min^−1^ mg^−1^ protein FW.

Catalase activity (CAT). CAT was assayed using a potassium phosphate buffer as per the protocol by Sairam et al. [35]. The assay involved monitoring the decrease in H_2_O_2_ concentration by measuring changes in light absorbance at 240 nm over 1 min. This was achieved by mixing 3.5 ml of H_2_O_2_ with 50 mL of deionized water (dH_2_O), then adding 70 mL of this mixture to a solution of 2.8 ml potassium phosphate buffer containing polyvinylpyrrolidone (PVP) and ethylenediaminetetraacetic acid (EDTA). Finally, 100 mL of the extract from the protein assay was added, and the absorbance was recorded at 240 nm after 1 min. The results were expressed in μmol min^−1^ mg^−1^ protein FW.

### Statistical analysis

The experimental setup for this study was structured as a factorial experiment, conducted following a completely randomized design (CRD) with three replications for each treatment. The data collected for various parameters were subjected to statistical analysis using MSTAT-C version 2.1 software. To discern significant differences between the means of different treatments, Duncan's multiple range test was employed. This statistical method was applied at two levels of error probability: five percent (0.05) and one percent (0.01). This approach ensured a rigorous and reliable analysis of the data, allowing for the identification of statistically significant effects of the treatments under study.

## Data Availability

Correspondence and requests for materials should be addressed to H.S.H.
